# ZINC40099027 Promotes Gastric Mucosal Repair in Ongoing Aspirin-Associated Gastric Injury by Activating Focal Adhesion Kinase

**DOI:** 10.3390/cells10040908

**Published:** 2021-04-15

**Authors:** Sema Oncel, Rashmi Gupta, Qinggang Wang, Marc D. Basson

**Affiliations:** 1 Department of Biomedical Sciences, University of North Dakota School of Medicine & Health Sciences, Grand Forks, ND 58203, USA; sema.oncel@und.edu; 2 Department of Surgery, University of North Dakota School of Medicine & Health Sciences, Grand Forks, ND 58203, USA; nfn.rashmi@und.edu (R.G.); qinggang.wang@und.edu (Q.W.); 3 Department of Pathology, University of North Dakota School of Medicine & Health Sciences, Grand Forks, ND 58203, USA

**Keywords:** FAK, migration, restitution, stomach, ulcer, mucosal healing, NSAIDs

## Abstract

Nonsteroidal anti-inflammatory drugs cause gastric ulcers and gastritis. No drug that treats GI injury directly stimulates mucosal healing. ZINC40099027 (ZN27) activates focal adhesion kinase (FAK) and heals acute indomethacin-induced small bowel injury. We investigated the efficacy of ZN27 in rat and human gastric epithelial cells and ongoing aspirin-associated gastric injury. ZN27 (10 nM) stimulated FAK activation and wound closure in rat and human gastric cell lines. C57BL/6J mice were treated with 300 mg/kg/day aspirin for five days to induce ongoing gastric injury. One day after the initial injury, mice received 900 µg/kg/6 h ZN27, 10 mg/kg/day omeprazole, or 900 µg/kg/6 h ZN27 plus 10 mg/kg/day omeprazole. Like omeprazole, ZN27 reduced gastric injury vs. vehicle controls. ZN27-treated mice displayed better gastric architecture, with thicker mucosa and less hyperemia, inflammation, and submucosal edema, and lost less weight than vehicle controls. Gastric pH, serum creatinine, serum alanine aminotransferase (ALT), and renal and hepatic histology were unaffected by ZN27. Blinded scoring of pFAK-Y-397 immunoreactivity at the edge of ZN27-treated lesions demonstrated increased FAK activation, compared to vehicle-treated lesions, confirming target activation in vivo. These results suggest that ZN27 ameliorates ongoing aspirin-associated gastric mucosal injury by a pathway involving FAK activation. ZN27-derivatives may be useful to promote gastric mucosal repair.

## 1. Introduction

Nonsteroidal anti-inflammatory drugs (NSAIDs) are among the most commonly prescribed medications for inflammatory, acute, and chronic pain, comprising 7.7% of worldwide prescriptions [1]. Sixty-five percent of NSAID-users develop upper or lower GI ulcers [2,3], and the annual medical costs of adverse GI events associated with NSAID use are likely to exceed $4 billion in the US, alone [4]. Although proximal gut NSAID-induced injury reflects cyclooxygenase (COX)-1 inhibition and increased mucosal permeability due to lysed phospholipids of mucosal epithelial cells, NSAID distal enteropathy is both real and has a very different pathogenesis [5,6]. Enterohepatic circulation of NSAIDs that complex with bile acids is the primary cause of lower GI injury [5,6,7]. Classic medical therapy for preventing or treating NSAID-induced ulcers involves proton pump inhibitors (PPIs) or histamine-2 receptor antagonists (H2-antagonists) [8], but these only reduce gastric acid secretion without directly promoting mucosal restitution. Furthermore, we are increasingly coming to understand that chronic PPI use may have side effects [9,10], such as bone fracture and infection [1]. In addition, although PPIs ameliorate upper GI injury, PPIs actually worsen NSAID enteropathy by suppressing gastric acid secretion and changing the enteric microbiome [1,6]. PPIs are, therefore, no longer recommended for use in this context [11]. Less potent antisecretory H2 blockers are likely to have similar but lesser issues. It would, therefore, seem desirable to identify a new agent that could treat both upper and lower GI injury. 

The nonreceptor tyrosine kinase focal adhesion kinase (FAK) promotes epithelial restitution by regulating focal adhesion assembly and disassembly and, thus, cell migration [12,13,14]. Inhibiting FAK inhibits migration [15]. The migrating cells adopt a squamous morphology and migrate across the lesion as a sheet [16,17,18]. While the proportion of activated FAK during epithelial sheet migration increases, the actual amounts of FAK and activated FAK397 are decreased in migrating GI cells in vitro [14] and at the edge of human gastric and colonic ulcers in vivo [19]. This makes FAK an attractive target for therapeutic intervention to promote mucosal healing. While screening molecules were chosen from the ZINC database that might inhibit FAK–Akt binding and FAK activation, we unexpectedly found a small molecule, ZINC40099027 (ZN27), that activates FAK [20]. Zn27 is a novel, potent, and selective activator acting allosterically on the 35 kDa FAK kinase domain [21]. Indeed, ZN27 activates FAK and promotes wound closure in mice after injury of the distal small bowel by a single high dose exposure to the NSAID indomethacin [22]. However, it remained unclear whether ZN27 would stimulate gastric mucosal healing and overcome ongoing NSAID injury. We, therefore, sought to investigate whether ZN27 would accelerate gastric epithelial monolayer wound closure in vitro by activating FAK and the efficacy of ZN27 in treating ongoing aspirin-associated gastric injury by promoting epithelial sheet migration in vivo.

We conducted preliminary studies in human and rodent gastric cell lines to confirm that Zn27 works in gastric epithelial cells and then studied the response to ongoing aspirin-associated gastric injury in mice, comparing the results of Zn27 treatment to that of treatment with omeprazole as a positive control. Our data suggest that small molecule ZN27 can promote gastric epithelial restitution in vitro and mucosal healing in mice by involving FAK activation.

## 2. Materials and Methods

### 2.1. Reagents

Dulbecco’s Modified Eagle’s medium (DMEM)/Ham’s F12 medium (#25-503) and RPMI-1640 medium (#25-506) were from Genesee Scientific (San Diego, CA, USA). Antibodies to FAK-Tyr-397 (#700255), 0.25% Trypsin-EDTA (#25200056), and Ham’s F-12K (Kaighn’s) medium (#21127-022) were from Thermo Fisher Scientific (Waltham, MA, USA). Secondary antibodies antirabbit 680 (#925-68073) and antimouse 800 (#925-32213) were from LI-COR (Lincoln, NE, USA). Ki-67 antibody (#9027) and SignalStain Boost Reagent (#8114) were from Cell Signaling (Danvers, MA, USA). Hydroxyurea (#H8627) and aspirin (#A2093) were obtained from Sigma Aldrich (St. Louis, MO, USA). Antibodies to FAK-Tyr-397(ab81298) and DAB substrate kit (ab64238) were from Abcam (San Francisco, CA, USA). We also used antibodies to total FAK (Anti-FAK, clone 4.47, 05-537, EMD Millipore, Temecula, CA, USA). ZINC40099027 was purchased from Enamine (Monmouth Junction., NJ, USA). The cell counting kit-8 (CCK-8) (#CK04-01) was purchased from Dojindo Molecular Technology (Rockville, MD, USA). Mouse alanine aminotransferase (ALT) ELISA kit (#MBS264717) was from MyBioSource (San Diego, CA, USA). Mouse creatinine kit (#80350) was from Crystal Chem (Elk Grove Village, IL, USA). Vectastain Elite ABC kit (#PK-6101) and biotinylated antirabbit secondary antibody (#PK-6101) were from Vector Laboratories (Burlingame, CA, USA). Omeprazole was purchased from Wedgewood Pharmacy (Swedesboro, NJ, USA). ZINC40099027 was purchased from Enamine (Monmouth Jct., NJ, USA). 

### 2.2. Cell Culture

We obtained RGM1 rat gastric epithelial cells (RCB0876) from the Riken BioResource Center (Saitama, Japan). NCI-N87 (CRL-5822) cells and AGS (CRL-1739) cells were obtained from the American Tissue Culture Collection (ATCC, Manassas, VA, USA). RGM1 cells were placed in a humidified incubator at 37 °C with 5% CO_2_ in Dulbecco’s modified Eagle’s medium (DMEM)/Ham F12 Medium with 100 U/mL penicillin, 100 μg/mL streptomycin, and 20% fetal bovine serum and cultured per Riken recommendations. NCI-N87 cells and AGS cells were maintained at 37 °C with 5% CO_2_ in RPMI-1640 and Ham’s F-12K medium, respectively, with 100 U/mL penicillin, 100 μg/mL streptomycin, and 10% fetal bovine serum. Both cell lines were cultured per ATCC recommendations.

### 2.3. Western Blotting

To study FAK activation in RGM1, AGS, and NCI-N87 cells, 80–90% confluent gastric epithelial cells were maintained in 1% heat-inactivated bovine serum albumin-coated bacteriologic plastic dishes to prevent adhesion-associated background FAK activation [22]. These suspended cells were treated with 10 nM ZN27 and DMSO (0.05%) for an hour before lysis for Western blotting in lysis buffer (50 mM Tris, 150 mM NaCl, 1 mM EDTA, 1 mM EGTA, 1% Triton-X-100, 1% deoxycholic acid, 0.1% SDS, 10% glycerol, aprotinin, and leupeptin). Protein concentrations were estimated by bicinchoninic acid assay. Proteins (40 µg) were then resolved by 10% SDS-PAGE, transferred onto nitrocellulose membranes, and blotted with antibody to Tyr-397-phosphorylated FAK (1:1000) and antirabbit 680 (1:20,000) as previously described, before quantitation using Kodak Scientific Imaging Systems 1D, V.3.5.4 [23]. Antibody to total FAK (1:1000) with a secondary antimouse 800 (1:20,000) served as a loading control. Densitometry was conducted on exposures within the linear range.

### 2.4. Wound Closure In Vitro 

RGM1, AGS, and NCI-N87 cells were seeded at 80% confluence into 6-well plates precoated with type I collagen type I [22,24]. After monolayers reached 100% confluence (48–72 h after seeding), they were wounded with nonbarrier autoclaved 1 mL pipette tips. Cells were then treated with either ZN27 or a DMSO vehicle control and cultured either without or with 5 mM hydroxyurea (HU) in serum-free media for 24 h to prevent proliferation. Wound images were captured using an inverted light microscope (OLYMPUS CK2, Center Valley, PA, USA) at 0 h and 24 h after wounding. Wound areas were measured with Image J software.

### 2.5. Drugs for In Vivo Use 

Aspirin was finely pulverized in a mortar and then prepared in a vehicle solution of 1% Tween 80 (well-tolerated in rodents orally) to reduce the surface tension and, therefore, to increase the stability of aspirin suspension for oral administration [25,26]. Aspirin was prepared freshly before each use and administered by gavage. ZN27 at 900 µg/kg or DMSO vehicle was dissolved in physiologic (0.9%) saline to make 100 µl volume and administered by intraperitoneal injection (ip). Omeprazole at 10 mg/kg and coconut oil were given by gavage. 

### 2.6. Animals

All animal procedures were approved by the University of North Dakota Institutional Animal Care and Use Committee under protocol number 1906-2. All mouse experiments were performed with 8–12-week-old C57Bl/6J male and female mice purchased from the Jackson Laboratory (Bar Harbor, ME, USA) or bred in-house at the University of North Dakota, with similar results. Mice were acclimatized to laboratory conditions for a week before starting the experiments. Mice were bred and housed in temperature-controlled rooms with a 12:12 h light–dark cycle at 23 ± 0.5 °C. 

### 2.7. Aspirin Lesion Induction and Experimental Design 

Aspirin-induced lesion formation was performed following the method of Seong-Soo Choi, 2012, and Szelenyi, 1978, with slight modifications [27,28]. The normal mouse stomach pH is around 3.0 and rises to 4.0 after fasting [29,30], and Dressman’s work shows that aspirin solubility is pH-dependent, increasing with increasing pH value above the pKa (3.5) of aspirin [31]. Therefore, briefly, in the ongoing aspirin-induced gastric injury model, 8–12 weeks old C57BL/6J mice were fasted for 12 h with free access to water to empty the stomach to increase gastric pH to facilitate gastric injury upon aspirin administration for the first day before the initial 300 mg/kg aspirin administration by gavage. For the remainder of the study (days 2–5), food and water were available ad libitum until the sacrifice day, and mice were administered aspirin for four more days after the initial dose. Bodyweight was measured daily over the five days of the treatment period. We chose a ZN27 dose and administration interval based upon our previous work [22]. To study ZN27 effects in an ongoing aspirin-induced gastric injury model, 24 h after the first administration of 300 mg/kg aspirin, DMSO (vehicle for ZN27) and 900 µg/kg ZN27 were given by intraperitoneal injection every 6 h, and 10 mg/kg omeprazole and coconut oil (vehicle for omeprazole) was administered in C57BL/6J mice by gavage once daily until day 6. Aspirin gavage administration took place in the morning and omeprazole gavage administration in the evening.

Male and female mice were randomly assigned to five groups. Group 1 (*n* = 9) did not receive any substances (including aspirin), serving as normal controls. All other groups received treatments in combination with aspirin. Group 2 (*n* = 12) received DMSO (vehicle control for ZN27) and coconut oil (vehicle control for omeprazole) and served as negative controls. Group 3 (*n* = 9) received omeprazole and DMSO, serving as positive controls. Group 4 (*n* = 18) received ZN27 and coconut oil and represented the test group. Group 5 (*n* = 8) received both omeprazole and ZN27 to study their combined effects. Supplementary Figure S1 summarizes the study design. 

At day 6, mice were anesthetized with isoflurane, and blood was drawn by cardiac puncture for assay of serum levels of creatinine and ALT. Animals were then sacrificed by cervical dislocation before removing the stomach, liver, and kidney for measurement of lesions and histological examination. The stomach was opened along the greater curvature. Stomach contents were gently collected to Eppendorf tubes for gastric pH measurements. Then stomachs were washed with phosphate-buffered saline to remove any contents before imaging. For each quadrant of the stomach, mucosal erosions (hyperemic areas) were imaged with an OLYMPUS Q Color 5 digital camera to determine a gross pathology score. The severity of gastric mucosal lesions was graded for each mouse with the Szelenyi method [28], modifying the scoring as (0) no erosion, (1) petechiae erosions, (2) erosions < 1 mm, and (3) erosions > 1 mm (See Supplementary Figure S2). Lesion scoring for each animal was calculated as follows: [(0 × number of lesion 0) + (1 × number of lesion 1) + (2 × number of lesion 2) + (3 × number of lesion 3)] [27,28]. All scorings were done by three different observers who were blinded to group assignments. All three observers found essentially similar trends (data not shown). One observer’s scoring is shown here. There was no difference between the genders; therefore we pooled male and female mice data for final analysis.

### 2.8. Hematoxylin and Eosin (H&E) Staining 

Mouse stomach, kidney, and liver tissues were fixed in 10% neutralized buffered formalin solution for 48 h and then transferred into phosphate-buffered saline at 4 °C for storage until processing as paraffin-embedded tissue blocks. Five-micron sections from the same area were stained with hematoxylin and eosin. Histologic slides were blindly reviewed by two independent specialists. Histological characterization comprised the following parameters for stomach: the presence of hemorrhage, inflammation, and thickness of each gastric layers; for kidney: tubular necrosis, inflammatory cells, glomeruli changes; for liver: vacuolated change, inflammatory cells, hepatitis, cholestasis, or fibrosis. All sections were examined entirety of by reviewers by use of an objective lens with a magnification of 20×. For histological stomach scoring, we followed Eaton’s protocol with slight modification [32]. Briefly, only fields that contained full-thickness gastric mucosa were scored. Each sample, located at the same distance from the forestomach, was divided into ten equal fields, then scored only for the presence or absence of the hyperemia or neutrophilic infiltration (polymorphonuclear leukocytes (PMN)), and the results were reported as the percentage of fields affected on each sample. For each sample, the number of positive fields was divided by the total number of fields (10), then multiplied by 100 to calculate the percentage of affected fields. The histologic criteria scored were defined as follows: for neutrophilic inflammation, we scored positive for the section if 10 or more neutrophils were in a cluster per 1000 µm^2^; for hyperemia, we scored positive for the section if there was any hyperemia/congestion. For the quantification of gastric layer lengths, each microscopic field was divided into ten equal sections, and the length of the gastric layer was measured from the middle of each section [33]. Then, the average length was calculated for each sample. The scoring systems used in this work allowed a quantitative assessment of both macroscopic and microscopic lesions, including the size and number of gastric erosions and the percentage of inflammation and hemorrhages.

### 2.9. Immunohistochemistry 

Stomach tissue sections were prepared for immunostaining as previously described [22]. Briefly, paraffin-embedded tissue sections were deparaffinized, rehydrated, and subjected to antigen retrieval by boiling in sodium citrate (pH 6.0) to expose target proteins. Sections were incubated with 0.3% hydrogen peroxide for 30 min at room temperature and then blocked with 10% normal goat serum. Tissue samples were then incubated for 1 h at room temperature with phospho-FAK(Tyr397) antibody (1:1000), followed by incubation with a biotinylated antirabbit secondary antibody and then incubation with an avidin–biotin complex reagent for 30 min at room temperature. Reactions were detected with 3,3′-diaminobenzidine solution. Then, sections were counterstained with hematoxylin. The intensity of immunoreactivity for FAK phosphorylation in the migrating epithelium at the edge of the lesion was scored by three blinded observers, with 0 being the least amount of detectable positive staining and 3 being the highest positive staining (See Supplementary Figure S3). All three observers found essentially similar trends (data not shown). One observer’s scoring is shown here. For Ki67 staining, we followed the Cell Signaling protocol. Briefly, after antigen unmasking, tissue sections were incubated with Ki67 antibody (1:100) overnight. On the next day, the sections were incubated with SignalStain Boost Reagent for 30 min and then counterstained with hematoxylin. For immunohistochemical quantification of Ki67 positive cells, we followed Ocampo’s calculations [34]. Briefly, three sections per tissue from four mice/group were analyzed. All quantifications were performed by an observer blinded to the group assignments.

### 2.10. Measurement of Gastric pH 

After the animals had been euthanized, their stomachs were removed and opened along the greater curvature. Approximately 20 µL of the fluid from the stomach was collected for gastric pH measurement with an Orion 2-Star pH benchtop meter from Thermo Scientific (Waltham, MA, USA). The pH meter had been previously calibrated at two points using standard solutions of pH 4.0 and pH 7.0.

### 2.11. Mouse Serum Creatinine and Alanine Aminotransferase (ALT) Levels

We measured mouse serum creatinine and serum alanine aminotransferase (ALT) levels by ELISA using assay kits purchased from Crystal Chem (Elk Grove Village, IL, USA) and MyBioSource (San Diego, CA, USA), respectively, and by following the manufacturer’s protocols. 

### 2.12. Statistical Analysis 

Data are represented as mean ± standard deviation of the mean. Data were normalized to the vehicle DMSO (0.05%) values in in vitro studies. Results were compared by Student’s t-test; one-way analysis of variance, followed by a post hoc test (Sidak’s Multiple Comparison Test); or two-way analysis of variance, followed by a post hoc test (Tukey’s Multiple Comparison Test), as appropriate, seeking 95% confidence. 

## 3. Results

### 3.1. ZN27 Activates FAK in Rat and Human Gastric Epithelial Cells

**Figure 1:**
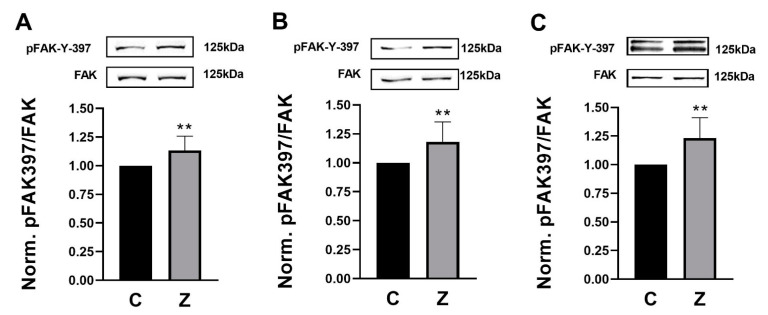
ZINC40099027 (ZN27) activates focal adhesion kinase (FAK) in rat (RGM1) and human (AGS and NCI-N87) gastric epithelial cells. Gastric epithelial cells were treated with 0.05% DMSO as a vehicle control or with 10 nM ZN27 for one hour. Total FAK served as a loading control. (**A**) Representative blots and FAK-Y-397/FAK fold change in RGM1 cells (*n* = 12, ** *p* < 0.01). (**B**) Representative blots and FAK-Y-397/FAK fold change in AGS cells (*n* = 10, ** *p* < 0.01). (**C**) Representative blots and FAK-Y-397/FAK fold change in NCI-N87 cells (*n* = 8, ** *p* < 0.01). Vehicle control–DMSO (C) and ZN27 (Z).

We treated rat (RGM1) and human (AGS and NCI-N87) gastric epithelial cells with 10 nM ZN27 at 37 °C for 1 h. ZN27 increased FAK-Tyr 397 phosphorylation, in comparison to DMSO controls in RGM1, AGS, and NCI-N87 cells, by 13.4 ± 12.4% (*n* = 12, ** *p* < 0.01) (Figure 1A), 18.2 ± 17.1% (*n* = 10, ** *p* < 0.01) (Figure 1B), and 23.2 ± 17.9% (*n* = 8, ** *p* < 0.01) (Figure 1C), respectively. These results demonstrate that ZN27 activates FAK in rat and human gastric epithelial cell lines. 

### 3.2. ZN27 Stimulates Monolayer Wound Closure in Rat and Human Gastric Epithelial Cells

**Figure 2:**
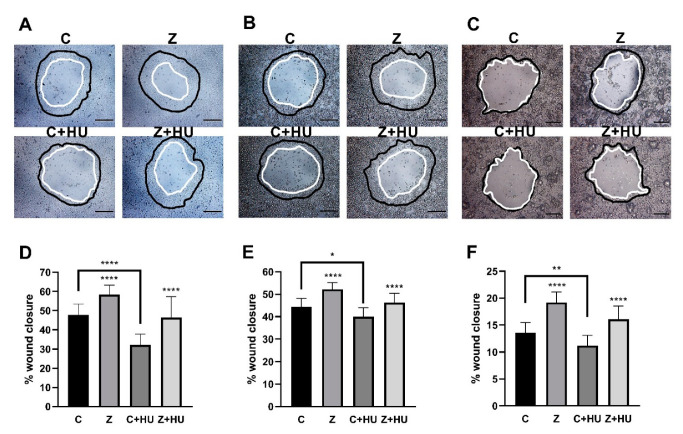
ZN27 stimulates rat (RGM1) and human (AGS and NCI-N87) gastric epithelial cell monolayer wound closure. (**A**–**C**) Typical wound images for RGM1, AGS, and NCI-N87 cells, respectively, treated with DMSO, ZN27 (10 nM), DMSO + Hydroxyurea (HU, 5 mM), or ZN27 (10 nM) + HU (5 mM) in serum-free media. (**D**) ZN27 at 10 nM accelerates circular wound closure in RGM1 cell monolayers on collagen I, even when proliferation is blocked by 5 mM hydroxyurea (*n* = 12–18, pooled from 6 separate studies with similar results, **** *p* < 0.0001). (**E**) ZN27 at 10 nM accelerates circular wound closure in AGS cell monolayers on collagen I, even when proliferation is blocked by 5 mM hydroxyurea. (*n* = 12–16, pooled from 6 separate studies with similar results, **** *p* < 0.0001, * *p* < 0.05). (**F**) ZN27 at 10 nM accelerates circular wound closure in NCI-N87 cell monolayers on collagen I, even when proliferation is blocked by 5 mM hydroxyurea. (*n* = 16–18, pooled from 6 separate studies with similar results, **** *p* < 0.0001, ** *p* < 0.01). The presence of 5 mM hydroxyurea prevented proliferation-related wound closure and, therefore, reduced overall wound closure in both cell lines. Black represents the circumference of the 0-h wounds; white represents the circumference of the wounds 24 h later. Vehicle control–DMSO (C), ZN27 (Z), hydroxuyurea (HU). All images were captured at 10x magnification. Scale bars, 200 µm.

We next studied the effect of 10 nM ZN27 on monolayer wound closure on rat (RGM1) and human (AGS and NCI-N87) gastric epithelial cells in the presence and absence of hydroxyurea in serum-starved media for 24 h to prevent proliferation (Figure 2A–C, respectively). Preliminary studies suggested that cell proliferation was blocked by 5 mM hydroxyurea in all gastric epithelial cells studied (See Supplementary Figure S4). 

ZN27 stimulated gastric epithelial monolayer wound closure by 21.9 ± 10.3% (*n* = 18, **** *p* < 0.0001) (Figure 2D), 17.6 ± 6.8% (*n* = 12, **** *p* < 0.0001) (Figure 2E), and 42.0 ± 14.4% (*n* = 16, **** *p* < 0.0001) (Figure 2F), compared to wounded monolayers treated with the DMSO vehicle, alone, in RGM1, AGS, and NCI-N87 cells, respectively. The presence of 5 mM hydroxyurea prevented proliferation-related wound closure and, therefore, reduced overall wound closure from 47.8 ± 5.5% (*n* = 18) to 32.2 ± 5.5% (*n* = 12, **** *p* < 0.0001) (Figure 2D), 44.4 ± 3.7% (*n* = 12) to 40.0 ± 4.0% (*n* = 16, * *p* < 0.05) (Figure 2E), and 13.5 ± 5.9% (*n* = 18) to 11.2 ± 1.4% (*n* = 16, ** *p* < 0.01) in DMSO-treated monolayer wounds in RGM1, AGS, and NCI-N87 cells, respectively. However, ZN27 still promoted monolayer wound closure, even in the presence of hydroxyurea (5 mM), in comparison to DMSO control cells in the presence of hydroxyurea, by 46.3 ± 11.0% (*n* = 12, **** *p* < 0.0001) (Figure 2D) in RGM1 cells, 46.3 ± 4.1% (*n* = 16, **** *p* < 0.0001) (Figure 2E) in AGS cells, and 16.1 ± 2.5% (*n* = 18, **** *p* < 0.0001) (Figure 2F) in NCI-N87 cells. 

### 3.3. ZN27 Promotes Gastric Mucosal Healing in Ongoing Aspirin-Induced Gastric Injury

**Figure 3:**
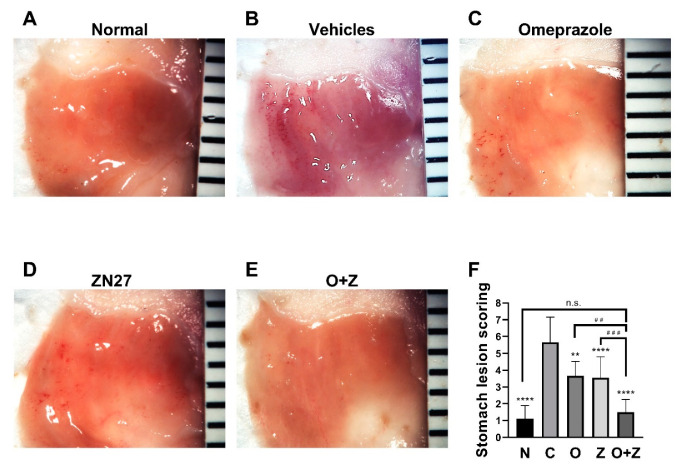
ZN27 promotes the healing of the aspirin-induced gastric injury in mice. Representative images of gastric lesions in mice receiving no treatment (**A**), vehicles (**B**), omeprazole (**C**), ZN27 (**D**), or omeprazole/ZN27 (**E**) at day 6, respectively. (**F**) Gastric lesion scoring in mice receiving no treatment (*n* = 9), omeprazole (*n* = 9), ZN27 (*n* = 18), or omeprazole/ZN27 (*n* = 8) are compared with gastric lesion scoring from vehicles-treated mice (*n* = 12) at day 6 (** *p* <0.01, **** *p* < 0.0001). Gastric lesion scoring in mice treated with Zn27, alone (*n* = 18), and omeprazole, alone (*n* = 9), are compared with lesion scores from mice treated with omeprazole and ZN27 together (*n* = 8) at day 6 (^##^ *p* < 0.01, ^###^ *p* < 0.001). Normal (*N*), vehicles (C), omeprazole (O), ZN27 (Z), and omeprazole/ZN27 (O + Z). (n.s. means not statistically significant.)

Having established that FAK is activated and migration is promoted in gastric epithelial cells, we next turned our attention to in vivo studies. Mice were treated with aspirin (300 mg/kg) daily for five consecutive days to induce gastric lesions. Representative images of aspirin-induced gastric lesions at day 6 are shown in Figure 3. Gross stomach images revealed that the stomach of normal mice displayed normal mucosa without hemorrhages (Figure 3A), whereas oral administration of aspirin-induced severe hemorrhagic streaks on the mucosal surface of the glandular stomach were observed in the vehicles (negative) control group (Figure 3B). 

The positive control omeprazole and the experimental ZN27 treatment similarly markedly reduced gross hemorrhages, in comparison to the negative vehicles control group (Figure 3C,D, respectively). Moreover, co-administration of omeprazole and ZN27 further reduced these hemorrhages (Figure 3E). Lesion scoring of images by observers blinded to the group assignments validated these gross impressions. Four days of omeprazole (10 mg/kg) (3.67 ± 0.87, *n* = 9, ** *p* < 0.01), Zn27 (900 µg/kg) (3.56 ± 1.25, *n* = 18, **** *p* < 0.0001), and omeprazole (10 mg/kg)/ZN27 (900 µg/kg) (1.50 ± 0.76, *n* = 8, **** *p* < 0.0001) treatments substantially reduced the gastric lesions in mice, in comparison to aspirin/vehicle-treated control mice (5.67 ± 1.50, *n* = 12) (Figure 3F). Moreover, mice treated with both omeprazole (10 mg/kg) and ZN27 (900µg/kg) (1.50 ± 0.76, *n* = 8) had significantly reduced gastric lesion scores, in comparison to mice treated with either Zn27 (900µg/kg), alone (3.56 ± 1.25, *n* = 18, ^###^ *p* < 0.001), or omeprazole (10 mg/kg), alone (3.67 ± 0.87, *n* = 9, ^##^ *p* < 0.01). Indeed, lesion scores for mice receiving the combined treatment (1.50 ± 0.76, *n* = 8, *p* > 0.05) did not differ significantly from normal mice (1.11 ± 0.78, *n* = 9) (Figure 3F). These results suggest that ZN27 promotes gastric mucosal healing in aspirin-induced gastric injury in C57BL/6J mice, and omeprazole/ZN27 treatment further accelerates gastric mucosal healing. 

### 3.4. ZN27 Increases Mucosal Immunoreactivity for FAK-Y-397 but Did Not Change Immunoreactivity for Ki67 in Aspirin-Injured Gastric Mucosa

**Figure 4:**
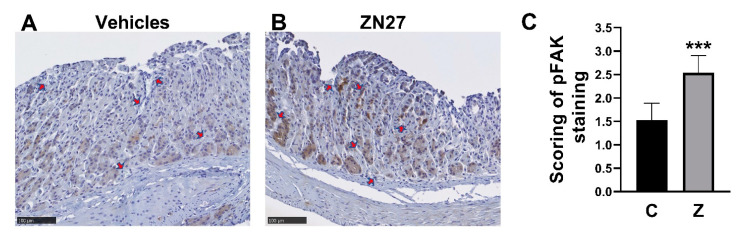
ZN27 increases the immunoreactivity of FAK-Y-397 in the aspirin-induced gastric injury model. (**A**) Representative immunohistochemical images of FAK-Y-397 phosphorylation at the edge of gastric lesions in mice receiving vehicles. (**B**) Representative immunohistochemical images of FAK-Y-397 phosphorylation at the edge of gastric lesions in mice receiving ZN27. Dark brown staining indicates FAK-Y-397 immunoreactivity. Arrows indicate gastric lesions (hyperemia). Scale bars, 100 µm. (**C**) Blindly scored immunoreactivity for FAK-Y-397 increases in ZN27 treated lesions (*n* = 7) vs. vehicle-treated lesions (*n* = 5), (*** *p* < 0.001). Vehicles (C), ZN27 (Z).

We next sought to evaluate whether FAK was, in fact, activated FAK at the edge of the lesions in ZN27-treated mice. Blindly scored immunohistochemical staining for phosphorylated FAK at the edge of gastric lesions demonstrated higher immunoreactivity in the mucosa at the edge of lesions from ZN27-treated mice (2.54 ± 0.36, *n* = 7, *** *p* < 0.001) than at the edge of lesions from vehicles-treated mice (1.53 ± 0.36, *n* = 5) on a 0–3 scale, as assessed by observers blinded to group assignment (Figure 4A–C). We also examined whether ZN27 has affected mucosal proliferation by staining for Ki67. We found no differences in Ki67 gastric mucosal immunoreactivity between the vehicle-treated mice and the ZN27-treated mice in the aspirin-induced murine gastric injury model (See Supplementary Figure S5).

### 3.5. ZN27 Decreases Hyperemia and Inflammatory Cell Infiltration and Ameliorates the Thickness of Gastric Layers in the Ongoing Aspirin-Induced Gastric Injury Model

**Figure 5:**
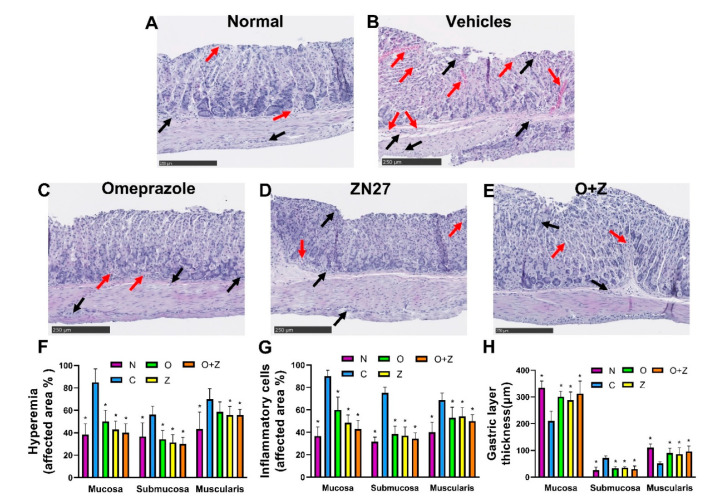
ZN27 decreases hyperemia and inflammatory cells and ameliorates the thickness of gastric layers in the ongoing aspirin-induced gastric injury model. Typical subsets of histologic fields that were scored positive for inflammatory cells and hyperemia/congestion in mice receiving no treatment (**A**), vehicles (**B**), 900µg/kg ZN27 (**C**), 10 mg/kg omeprazole (**D**), or omeprazole plus ZN27 (**E**) at day 6. Black arrows in the histologic images indicate inflammatory cells, and red arrows indicate hyperemia. For illustrative purposes, only partial fields are shown. The scale bar represents 250 µm. (**F**,**G**) Microscopic hyperemia and inflammatory cell infiltration scoring, respectively, in mice receiving vehicles (*n* = 8), ZN27 (*n* = 7), omeprazole (*n* = 7), or omeprazole plus ZN27 (*n* = 7) are compared with normal mice (*n* = 6) at day 6, assessed morphometrically in 10 sections/mouse by a blinded observer. (**H**) Gastric layer thickness in mice receiving vehicles (*n* = 8), ZN27 (*n* = 7), omeprazole (*n* = 7), or omeprazole plus ZN27 (*n* = 7) are compared with normal mice (*n* = 6) at day 6, assessed morphometrically in 10 sections/mouse by a blinded observer. * *p* < 0.05 vs. aspirin-injured vehicle controls. Scale bars, 250 µm. Normal (N), vehicles (C), omeprazole (O), ZN27 (Z), and omeprazole/ZN27 (O + Z).

Gastric tissues were stained with hematoxylin and eosin to evaluate the morphology of the gastric mucosa, submucosa, and muscularis. There was a strong correlation between the macroscopic and microscopic findings. The stomach of normal mice displayed few hyperemic areas and inflammatory cells, while exhibiting an intact mucosa with normal thickness of each gastric layers (Figure 5A). 

In contrast, the negative control (vehicles only) group that had been treated with aspirin but neither Zn27 nor omeprazole was characterized by distinctive injuries with extensive inflammatory cells, hyperemic areas, congestion, and severe alterations in each gastric layer, such as decreased mucosal thickness due to erosion of surface epithelial cells, increased submucosal thickness resulting from augmenting edema, and shrunk muscular thickness caused by compression from submucosal edema (Figure 5B). As can be seen in Figure 5C–E, less hyperemia, congestion, and fewer infiltrating inflammatory cells were observed in tissues from mice treated with 900 µg/kg ZN27, 10 mg/kg omeprazole, or the omeprazole (10 mg/kg)/ZN27 (900 µg/kg), in comparison to the negative control mice group receiving only aspirin and vehicles. Although both omeprazole and ZN27 decreased inflammatory cell infiltration, the inflammatory infiltrate in mice receiving ZN27 was significantly less than that in omeprazole-treated mice. The combination of omeprazole and ZN27 did not result in a statistically significant further decrease in the inflammatory infiltrate. The ZN27, omeprazole, and ZN27/omeprazole groups had notably thicker mucosa and muscularis and decreased submucosal thickness, more closely resembling the normal tissue (Figure 5C–H).

### 3.6. ZN27 Does Not Alter the Gastric pH in the Ongoing Aspirin-Induced Gastric Injury Model 

**Figure 6:**
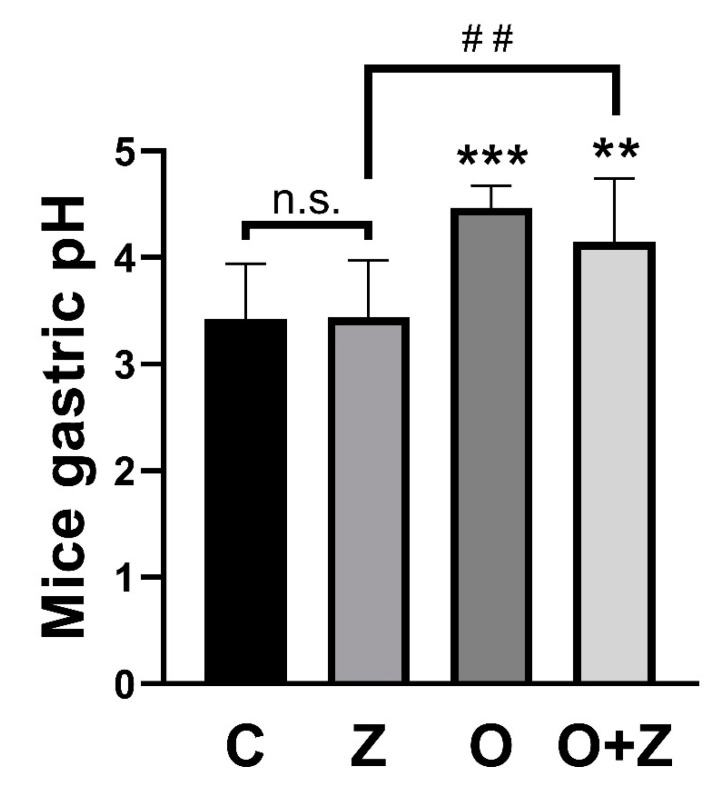
ZN27 does not change the gastric pH in the ongoing aspirin-induced gastric injury model. The gastric pH in the ZN27 (*n* = 10, *p* > 0.05) group does not change, in comparison to the vehicle control (*n* = 12) group. Omeprazole (*n* = 9, *** *p* < 0.001) and omeprazole/ZN27 (*n* = 8, ** *p* < 0.01) treatments increase gastric pH, in comparison to the vehicle control (*n* = 12) group. The gastric pH after omeprazole/ZN27 treatment (*n* = 8, ^##^ *p* < 0.01) is significantly higher than in the ZN27 treated (*n* = 10) group. Vehicle (C), omeprazole (O), ZN27 (Z), and omeprazole/ZN27 (O + Z). (n.s. means not statistically significant.)

The normal mouse gastric pH has been reported to be approximately 3 and to rise to 4 after fasting [29,30]. In our in vivo studies, gastric pH was significantly increased in animals receiving either omeprazole (4.5 ± 0.20, *n* = 9, *** *p* < 0.001) or omeprazole/ZN27 (4.1 ± 0.44, *n* = 8, ** *p* < 0.01) but not in animals receiving ZN27, alone (3.4 ± 0.53, *n* = 10, *p* > 0.05), when compared to animals receiving vehicle controls (3.4 ± 0.52, *n* = 12) (Figure 6).

### 3.7. ZN27 Treatment Ameliorates the Weight Loss Associated with Ongoing Aspirin-Induced Gastric Injury, Does Not Affect Mice Serum Creatinine or Alanine Aminotransferase, and Does Not Obviously Alter Kidney and Liver Morphology

**Figure 7:**
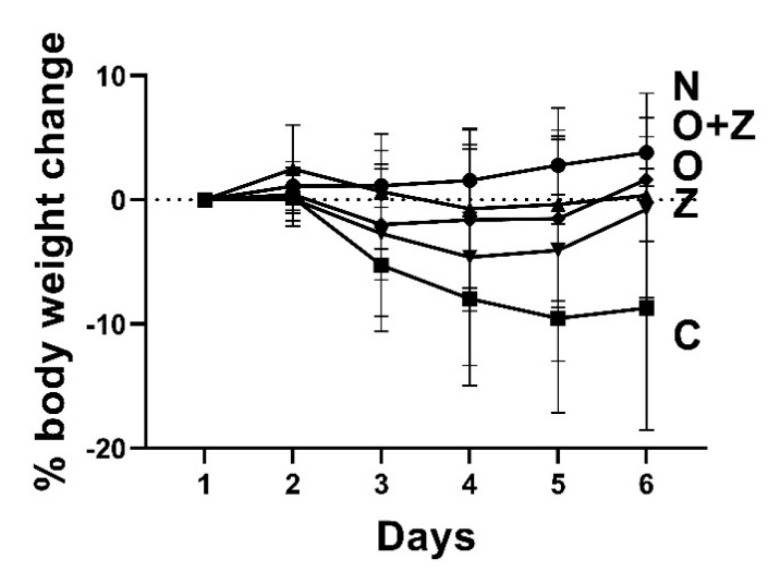
ZN27 treatment accentuates the weight loss associated with the ongoing aspirin-induced gastric injury. Y-coordinate indicates the percentage of the body weight change. The vehicle control group lost substantial weight. (*n* = 13) However, the changes in the omeprazole-treated group (*n* = 9), ZN27-treated (*n* = 18), and omeprazole/ZN27-treated (*n* = 8) groups were not significant, in comparison to the normal (*n* = 9) group (uninjured, untreated) by the end of the study. There were also no significant differences among the omeprazole, ZN27, and omeprazole/ZN27 groups. Normal (N), vehicle (C), omeprazole (O), ZN27 (Z), and omeprazole/ZN27 (O + Z).

Although normal (uninjured, untreated) mice gained weight slightly, the vehicle control group, injured by aspirin, exhibited substantial weight loss at the end of the study (−8.7 ± 9.3%, *n* = 12, *** *p* < 0.001) (Figure 7). Omeprazole-treated, ZN27-treated, and omeprazole/ZN27-treated groups displayed slight weight loss initially, which might reflect the aspirin injury, but by the end of the study, they began to gain the weight back. Thus, by the end of the study, they were not statistically different from the normal, uninjured mice.

Histological assessment of renal tissues revealed no obvious structural alterations between the normal group and the ZN27-treated group. We observed no evidence of tubular necrosis or infiltrating inflammatory cells, and the glomeruli appeared similar between the groups. In addition, serum creatinine in all groups was within the normal range (0.06–16 mg/dL, Crystal Chem) and did not differ between the normal group and the other treatment groups (Table 1). 

Histological assessment of hepatic tissues also showed no obvious structural alterations between the normal group and the ZN27-treated group. Vacuolated changes, inflammatory cells, hepatitis, cholestasis, or fibrosis were not observed. Moreover, although omeprazole/ZN27-treated mice had slightly increased ALT levels, in comparison to normal mice (9.17 ± 1.10 U/L vs. 6.53 ± 1.48 U/L, respectively), the serum ALT levels were well within the normal range (7.63–53.1 U/L, MyBioSource (San Diego, CA, USA)) for all groups (Table 1).

## 4. Discussion

NSAIDs are a common cause of mucosal injury in the stomach, as well as in the intestine. PPIs ameliorate proximal GI injury from NSAIDs but actually worsen distal GI injury by changing the gastric pH and, thus, the enteric microbiome [1,6]. The novel small molecule ZN27 activates FAK [20] by acting allosterically on the 35 kDa kinase domain of FAK [21] and promotes mucosal healing in acute indomethacin-induced distal small bowel injury in mice [22]. However, the effect of ZN27 on upper GI and ongoing injury had not been previously studied. This study demonstrates that ZN27 activates FAK in injured gastric mucosa and promotes gastric epithelial wound healing in vitro and in vivo, improving the gross and histological appearance of the gastric mucosa without changing gastric pH and with no obvious renal or hepatic toxicity in the ongoing aspirin-induced gastric injury. Combining omeprazole and ZN27 treatment yields an even better effect than either molecule, alone.

Having previously reported that ZN27 can promote mucosal restitution in acute (one dose) indomethacin-induced intestinal injury [22], we chose an ongoing aspirin-induced gastric injury model to investigate the effect of ZN27 for two reasons: First, gastric biology is different from intestinal biology. Indeed, NSAIDs injure the proximal and distal GI tract by distinct mechanisms [5,6,7]. COX-1 inhibition by NSAIDs is associated with damage to the gastric mucosal barrier with decreased bicarbonate and mucus secretion and increased gastric acid back diffusion, due to reduced blood flow, causing proximal GI injury [35,36]. However, distal small bowel injury is caused by NSAIDs complexing to bile acids and, therefore, potentiating the ability of these bile acids to damage the gut mucosa, as well as by alterations in the numbers and types of enteric bacteria [7]. Second, the risk of GI adverse effects with NSAIDs may depend on the dose and the duration of therapy [37,38]. Healing an ongoing injury is more challenging than healing a fresh wound that has previously occurred, due to, among other issues, differences in inflammatory responses [39,40]. For example, the host response continues with persistent recruitment of inflammatory cells to the injured area, and further damage ensues in ongoing injury, whereas the cause of the injury will be eliminated and repair will take place in an acute injury [39,40]. In addition to systemic damage, some NSAIDs, especially those of acidic nature like aspirin, directly damage the layer of phospholipids on the mucosal surface, rendering the mucosa less able to resist damage induced by luminal acid [5]. This leads to the infiltration of erosive gastric fluid into the mucosa, causing further inflammation and mucosal injury. This mechanism potentiates the toxicity of NSAIDs in the small intestine, because the enterohepatic circulation of these NSAIDs leads to repeated exposure of the intestinal epithelial cells to these drugs [5]. The current study shows that ZN27 treatment ameliorates the gross morphology of injured gastric tissues to the same extent as omeprazole in the setting of ongoing aspirin-induced gastric injury, suggesting that ZN27 derivatives could be used to treat both upper and lower GI injuries, whether from an acute event or a more tenacious GI injury associated with ongoing NSAID use. 

FAK regulates fundamental processes, such as cell adhesion, migration, apoptosis, and cell survival [41,42]. FAK activation is crucial for FA assembly and disassembly at the leading and trailing edges of migrating cells [42] and, thus, cell migration [12,13,14]. Although the proportion of FAK that is activated increases during epithelial sheet migration, the actual amounts of FAK and activated FAK-Y-397 are decreased in migrating GI cells in vitro [15] and at the edge of human gastric and colonic ulcers in vivo [43], due to decreases in FAK synthesis, potentially driven, at least in part, by changes in Transforming Growth Factor beta [44,45,46] as an unfortunate aspect of the migratory phenotype. Indeed, epithelial FAK levels vary substantially between the ulcer edge and adjacent tissue [43,45], which makes FAK an attractive target for a new generation pharmacotherapy to promote mucosal restitution [46]. FAK inhibition slows epithelial sheet migration [15] and, thus, prevents the stimulation of monolayer wound closure by ZN27 in Caco2 cells [22]. Stimulation of wound closure could be due to increased proliferation or increased migration or both. This study indicates that ZN27 accelerates FAK activation and wound healing in vivo and in vitro in murine and human gastric epithelial cells, even when we prevented proliferation with hydroxyurea in serum-free media. Furthermore, we found no difference in gastric mucosal proliferation, as measured by Ki67 immunoreactivity after ZN27 treatment. These results suggest that ZN27 promotes monolayer wound closure by increasing migration rather than stimulating proliferation, parallel with our previous in vitro and in vivo data [22,23]. These findings suggest that FAK activation by ZN27 and its promotion of mucosal healing are effective in the stomach, as well as the distal small bowel. 

The reduction in inflammatory cell infiltration that we observed in the gastric wall after ZN27 treatment could reflect the general recovery of the gastric barrier by increased mucosal restitution or some previously unknown anti-inflammatory properties of ZN27 or both. Omeprazole-treated mice also displayed a decrease in inflammatory infiltration, and, indeed, recent studies suggest that PPIs exert anti-inflammatory effects independently of their effects on gastric acid secretion [47,48]. However, ZN27 reduced this inflammatory infiltration more than omeprazole, even though their effect on mucosal healing seemed similar, suggesting a possible anti-inflammatory effect for ZN27, as well. The involvement of FAK in the inflammatory response is controversial. Some studies suggest that FAK mediates the release of proinflammatory cytokines, such as TNF-α and IL-1β [49], and may contribute to the worsening of inflammation. However, others suggest that nuclear FAK regulates the expression of some inflammatory genes, such as IL-33 and GATA4, resulting in reduced inflammatory responses by inducing regulatory T cells (T_reg_) and, thus, enhancing repair [50,51]. Indeed, IL-33 has an important role in the resolution of inflammation and repair of tissue damage, especially in GI mucosa [52]. If FAK activation does have an anti-inflammatory effect, this could reflect FAK activity within the injured tissue or within the inflammatory cells, themselves. FAK is also known to influence the release of chemotactic and inflammatory cytokines from epithelial cells, endothelial cells, and fibroblasts. In epithelial cells, FAK regulates downstream signals of IL-33 [53] and CXCL-8 [54] and inhibits GATA4 expression [55,56], thus reducing the inflammatory responses. On the other hand, in endothelial cells, FAK enhances MCP-1 release [57] and endothelial permeability [58], thus increasing the inflammatory response. Similarly, FAK modulates IL-1 [59], IL-6 [60], and IL-8 [60], which are involved in the initiation of pro-inflammatory responses in fibroblasts. Conversely, FAK also plays an important role within the inflammatory cells, themselves. For instance, FAK serves as an important node to regulate T-cell function by inhibiting the T-cell receptor function [61,62]. In addition, inhibition of FAK by extracellular pressure, such as that which may occur from local tissues edema, may enhance macrophage phagocytosis [63]. Likewise, FAK inhibition enhances the autophagic elimination of intracellular pathogens in macrophages, results in reduced infiltration of inflammatory cells, and reduces tissue damage [64]. Whether ZN27 is, in fact, anti-inflammatory and, if so, whether this reflects FAK activation in the inflammatory cells, themselves, FAK activation in the injured tissue with consequent alterations in chemotactic cytokine release or some other effect unrelated to FAK awaits further exploration beyond the scope of the current manuscript. 

It was noteworthy that ZN27 did not affect the gastric pH. PPIs are the most commonly co-prescribed drugs with NSAIDs to ameliorate NSAID-induced upper GI injury by reducing the secretion of gastric acid production [1]. However, they increase lower GI injury [65,66], at least in part by their effect on the intestinal microbiome, due to neutralization of the gastric pH [7,66] and are, therefore, no longer recommended for use in this context [11]. Indeed, many studies show that polypharmacy (NSAIDs, PPIs, and/or low-dose aspirin, etc.) markedly exacerbate NSAID-induced small intestinal injury, while gastric injury remained low [67,68]. In this study, ZN27, contrary to omeprazole, promoted mucosal epithelial healing without changing gastric pH and also without obvious weight loss or hepatic or renal toxicity. Furthermore, healing was accentuated when ZN27 and omeprazole were administered together. These findings suggest that ZN27 and omeprazole promote healing by different mechanisms. ZN27 derivatives could, thus, offer some clinical advantages over PPIs when treating NSAID-induced lower GI injury and might be useful alone or in combination with PPIs in treating acute gastric injuries. However, these complex treatment possibilities await further studies beyond the scope of the current manuscript. 

In summary, ZN27 ameliorates ongoing aspirin-induced gastric injury by activating FAK, promoting restitution, and alleviating hyperemia and inflammation in the stomach wall and does so without obvious toxicity. Taken together with our previous studies [21,22], these results raise the possibility that in the future derivatives of ZN27 could ultimately have therapeutic potential in either ongoing or acute NSAID-induced GI injuries, alone (for lower GI injury), or with PPIs (for upper GI injury). 

## 5. Patents

M.D.B. is a co-inventor on a currently pending patent application on the use of FAK activator (ZINC40099027) to promote mucosal healing.

## Figures and Tables

**Table 1:** ZN27 does not affect creatinine and alanine aminotransferase (ALT) levels in the ongoing aspirin-induced gastric injury model.

	Normal Range	Normal (*n* = 3)	Vehicles (*n* = 6)	Omeprazole (*n* = 5)	ZN27 (*n* = 6)	O + Z (*n* = 6)
Serum creatinine	0.06–16 mg/dL	0.11 ± 0.04	0.11 ± 0.03	0.09 ± 0.07	0.08 ± 0.03	0.11 ± 0.05
Serum ALT	7.63–53.1 U/L	6.53 ± 2.56	6.00 ± 2.02	6.11 ± 3.78	6.85 ± 1.90	9.17 ± 2.70

## Data Availability

The data used to generate the figures in this manuscript are available from the first author upon reasonable request.

## Supplementary Materials

The following are available online at https://www.mdpi.com/article/10.3390/cells10040908/s1, Figure S1: Experimental design for ongoing aspirin-induced stomach injury in mice. Figure S2: Erosion scoring table for the aspirin-induced gastric injury in mice, Figure S3: Scoring table for pFAK-Y-397 immunostaining in ongoing aspirin-induced injury in mice, Figure S4: Cell number change in rat and human gastric epithelial cells after hydroxyurea treatment, Figure S5: Immunostaining and quantification of Ki67 positive cells in mice stomach.
