# Supplementary figures and images for: ZINC40099027 Promotes Gastric Mucosal Repair in Ongoing Aspirin-Associated Gastric Injury by Activating Focal Adhesion Kinase
Source: Cells. 2021 Apr 15;10(4):908. doi: 10.3390/cells10040908 (PMC8071155; doi:10.3390/cells10040908)

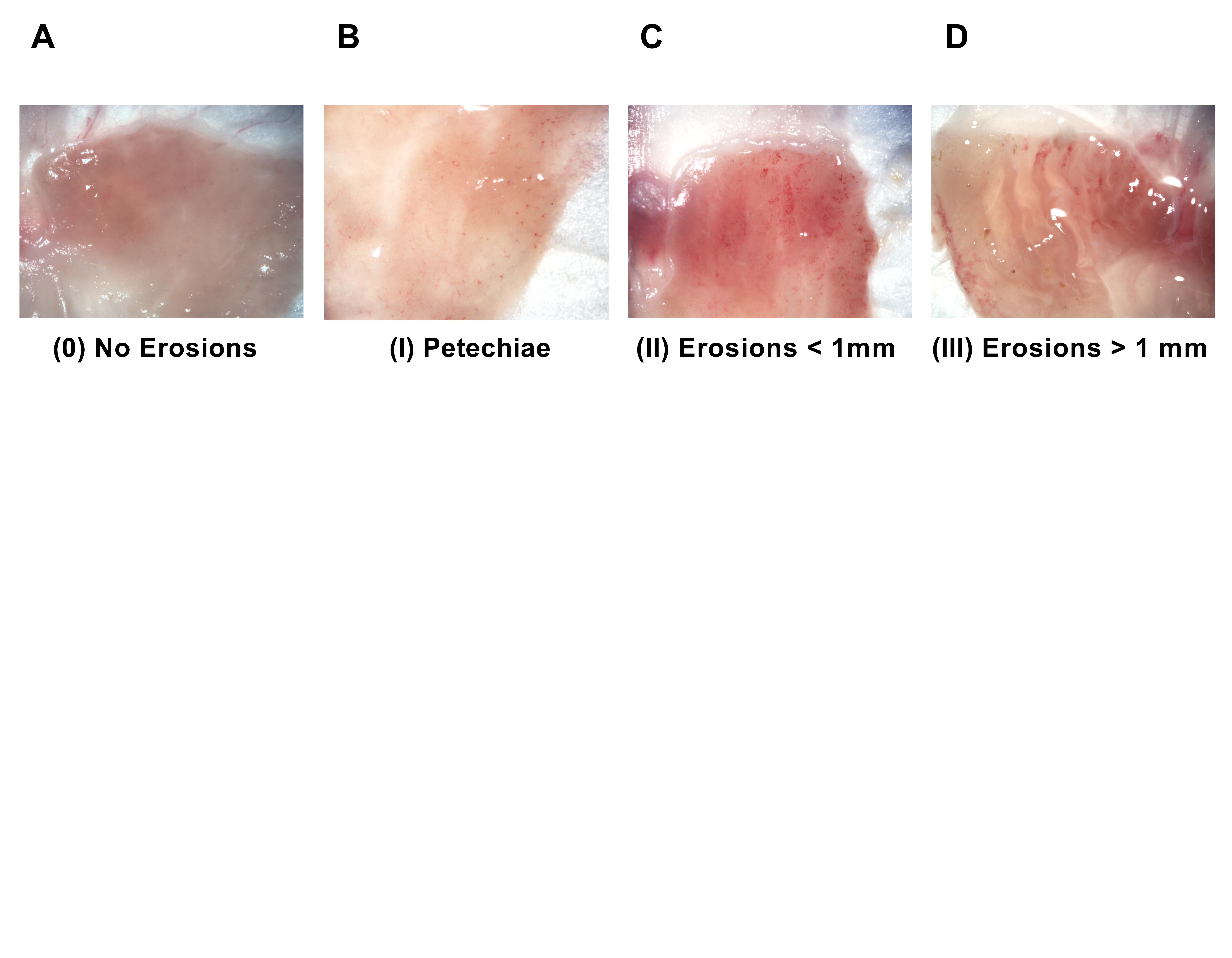

Supplement: Supplementary file 1 [file cells-10-00908-s001.zip › cells-1138664 sm/Supplementary Figure 2.jpg]

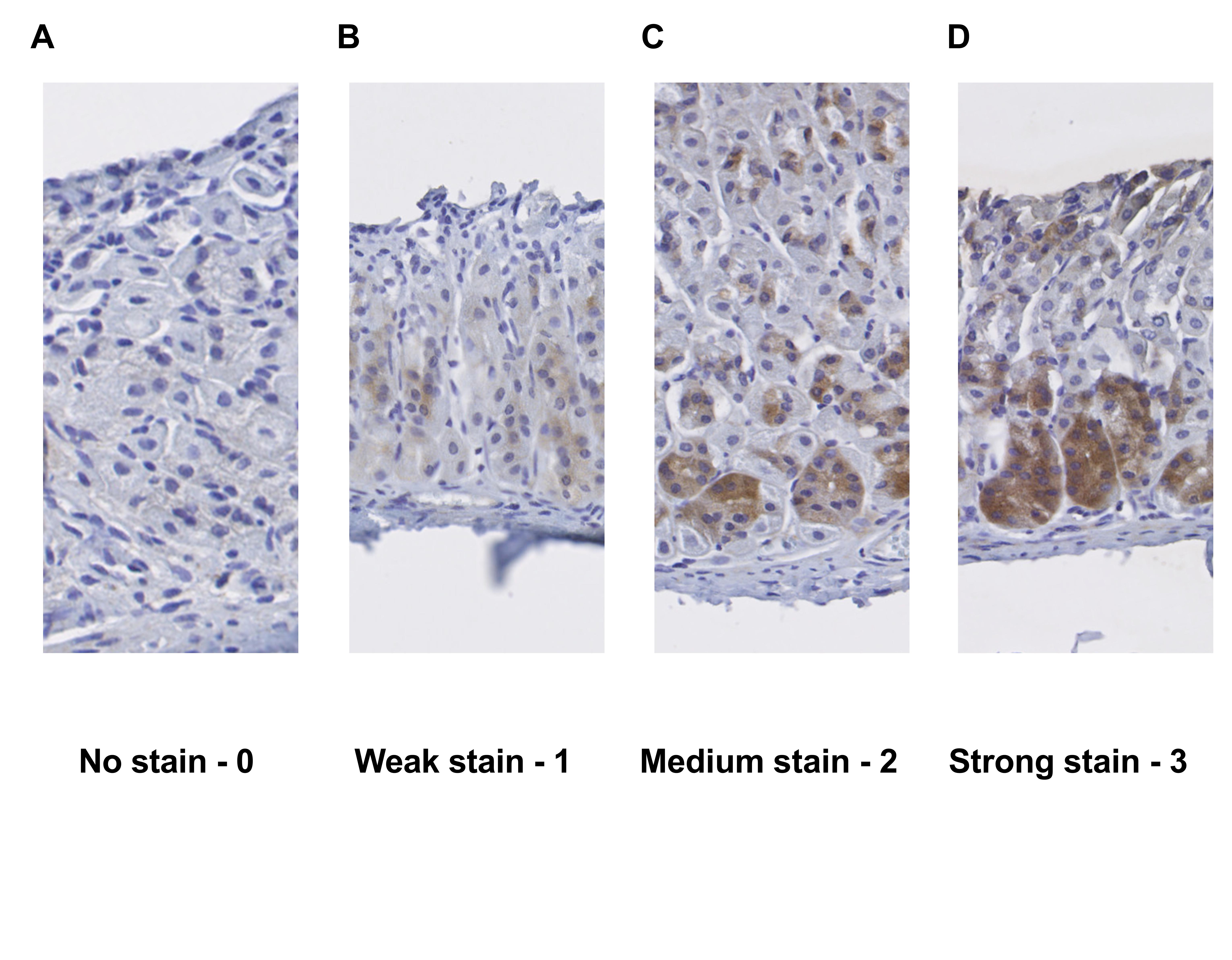

Supplement: Supplementary file 1 [file cells-10-00908-s001.zip › cells-1138664 sm/Supplementary Figure 3.jpg]

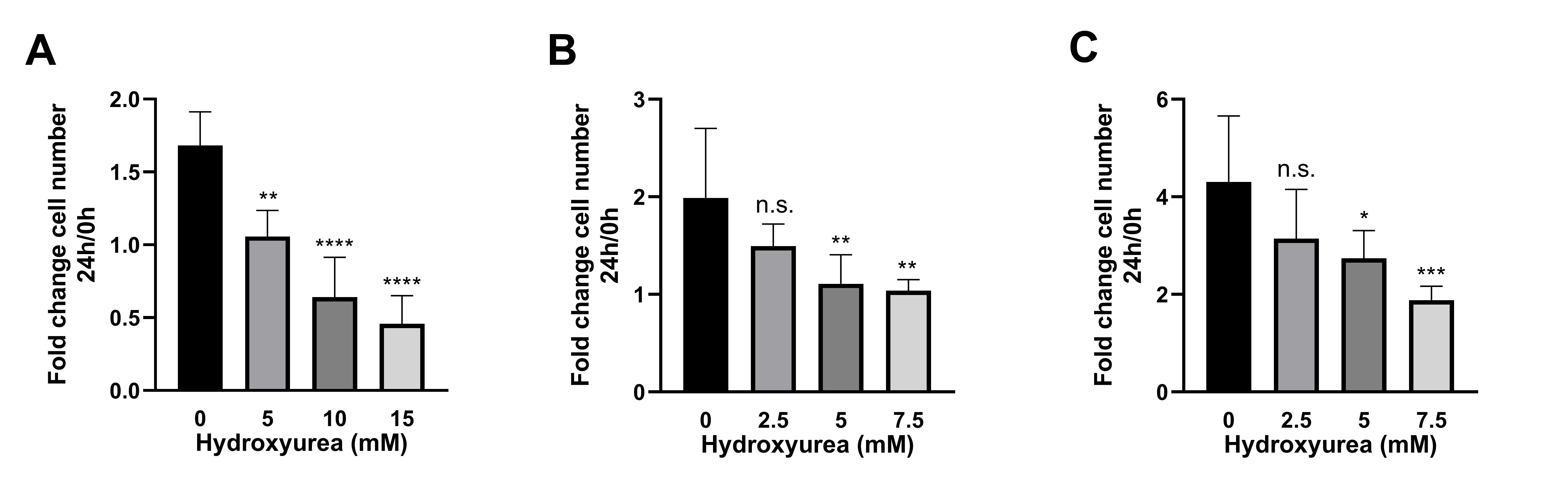

Supplement: Supplementary file 1 [file cells-10-00908-s001.zip › cells-1138664 sm/Supplementary Figure 4.jpg]

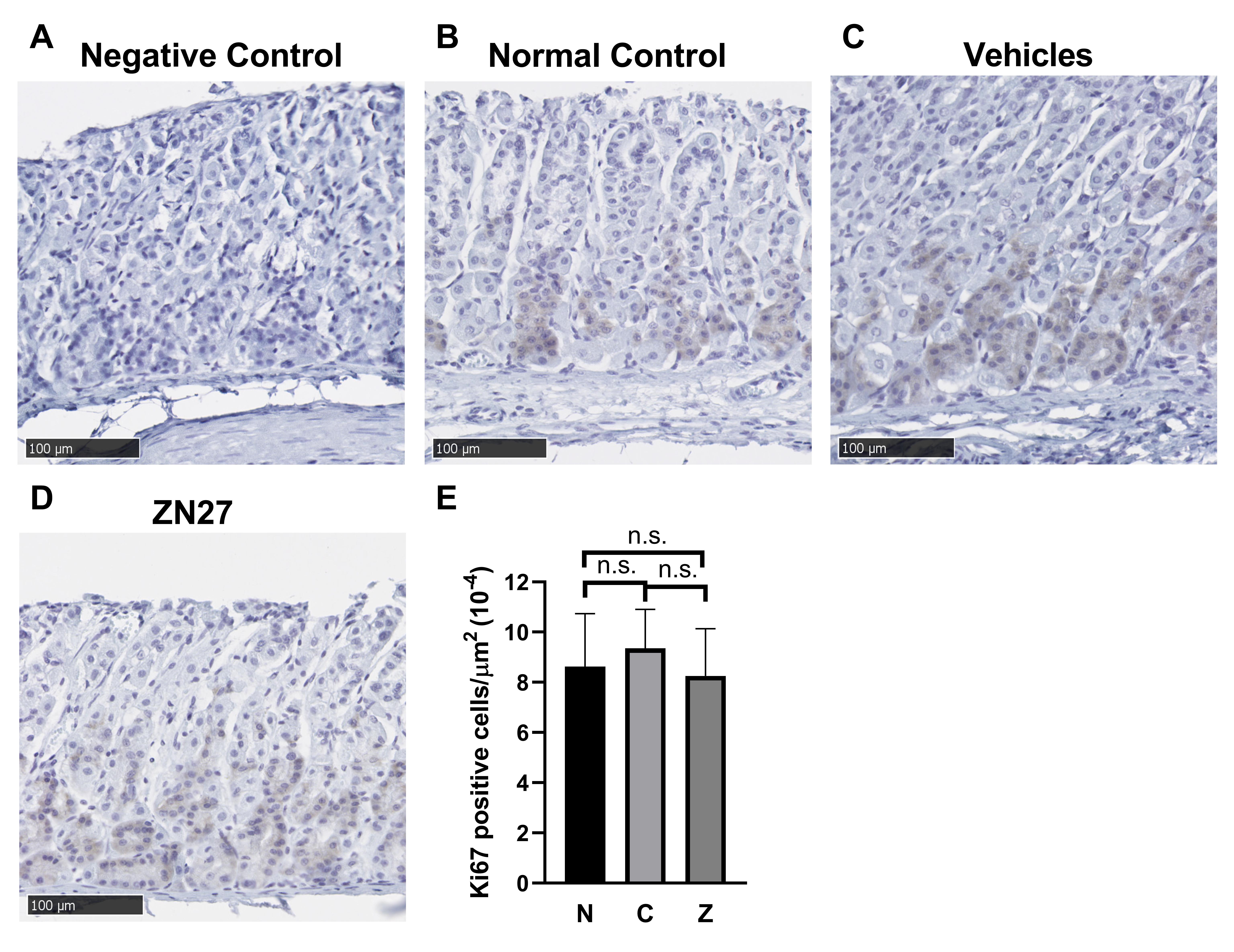

Supplement: Supplementary file 1 [file cells-10-00908-s001.zip › cells-1138664 sm/Supplementary Figure 5.jpg]
